# Adult Neural Stem Cells from Midbrain Periventricular Regions Show Limited Neurogenic Potential after Transplantation into the Hippocampal Neurogenic Niche

**DOI:** 10.3390/cells10113021

**Published:** 2021-11-04

**Authors:** Mareike Fauser, Kai F Loewenbrück, Johannes Rangnick, Moritz D Brandt, Andreas Hermann, Alexander Storch

**Affiliations:** 1 Department of Neurology, University of Rostock, Gehlsheimer Straße 20, 18147 Rostock, Germany; mareike.fauser@med.uni-rostock.de; 2 Division of Neurodegenerative Diseases, Department of Neurology, Technische Universität Dresden, Fetscherstraße 74, 01307 Dresden, Germany; kai.loewenbrueck@uniklinikum-dresden.de (K.F.L.); johannes.rangnick@posteo.de (J.R.); moritz.brandt@uniklinikum-dresden.de (M.D.B.); Andreas.Hermann@med.uni-rostock.de (A.H.); 3 German Center for Neurodegenerative Diseases (DZNE), Tatzberg 41, 01307 Dresden, Germany; 4 Translational Neurodegeneration Section, “Albrecht-Kossel”, Department of Neurology, University of Rostock, Gehlsheimer Straße 20, 18147 Rostock, Germany; 5 German Centre for Neurodegenerative Diseases (DZNE) Rostock-Greifswald, Gehlsheimer Straße 20, 18147 Rostock, Germany

**Keywords:** adult neural stem cells, adult neurogenesis, cell transplantation, neurogenic niche, dentate gyrus, non-neurogenic region, neuronal differentiation

## Abstract

The regulation of adult neural stem or progenitor cell (aNSC) proliferation and differentiation as an interplay of cell-intrinsic and local environmental cues remains in part unclear, impeding their role in putative regenerative therapies. aNSCs with all major properties of NSCs in vitro have been identified in a variety of brain regions beyond the classic neurogenic niches, including the caudal periventricular regions (PVRs) of the midbrain, though active neurogenesis is either limited or merely absent in these regions. To elucidate cell-intrinsic properties of aNSCs from various PVRs, we here examined the proliferation and early differentiation capacity of murine aNSCs from non-neurogenic midbrain PVRs (PVR_MB_) compared to aNSCs from the neurogenic ventricular-subventricular zone (PVR_V-SVZ_) 7 days after transplantation into the permissive pro-neurogenic niche of the dentate gyrus (DG) of the hippocampus in mice. An initial in vitro characterization of the transplants displayed very similar characteristics of both aNSC grafts after in vitro expansion with equal capacities of terminal differentiation into astrocytes and Tuj1^+^ neurons. Upon the allogenic transplantation of the respective aNSCs into the DG, PVR_MB_ grafts showed a significantly lower graft survival and proliferative capacity compared to PVR_V-SVZ_ transplants, whereby the latter are exclusively capable of generating new neurons. Although these differences might be—in part—related to the transplantation procedure and the short-term study design, our data strongly imply important cell-intrinsic differences between aNSCs from neurogenic compared to non-neurogenic PVRs with respect to their neurogenic potential and/or their sensitivity to neurogenic cues.

## 1. Introduction

Adult neurogenesis is a well-known phenomenon that occurs in two distinct germinal (neurogenic) niches of the rodent brain: the ventricular-subventricular zone (V-SVZ) within the periventricular regions of the lateral wall of the lateral ventricles (PVR_V-SVZ_) and the dentate gyrus (DG) of the hippocampus. Within the PVR_V-SVZ_, the cellular cascade leading to the formation of new neurons has been extensively studied with subependymal glial fibrillary acidic protein^+^ (GFAP^+^) astrocytes identified as the genuine adult neural stem cells (aNSCs). They generate—through various intermediate stages—distinct subtypes of interneurons determined to integrate into different cell layers in the olfactory bulb, where they are involved in olfactory learning processes (for a review, see [1]). The regulation of aNSC proliferation, migration and differentiation depends on both cell-extrinsic and cell-intrinsic mechanisms. The former consist of the neurogenic niche itself, with its highly specialized microenvironment and complex architecture of microglia, astrocytes, ependymal and vascular cells, acting as key cell-extrinsic regulators through the secretion of mitogens, e.g., brain-derived neurotrophic factor (BDNF) and neurotrophin-3 (NT-3) [2,3], regulation of signaling cascades involving e.g., SHH, BMP and Wnt [4,5,6] and neurotransmitter release [7,8,9]. Still, cell-intrinsic preconditions also seem to play a role in the regulation of PVR_V-SVZ_-olfactory bulb neurogenesis, e.g., in terms of positional specifications with different neuronal subtypes originating from distinct V-SVZ aNSCs along the rostro-caudal plane [10,11]. In addition, epigenetic regulation through chromatin modification [12], histone acetylation [13] and DNA methylation [14] seems equally important. 

Within the DG of the hippocampus, adult neurogenesis is a far more localized process, since the glutamatergic progeny of hippocampal aNSCs in the subgranular zone (SGZ) migrates only a short distance into the adjacent granular zone (GZ), where it plays an important role in pattern separation as a distinct process within memory formation [15]. Hippocampal aNSCs expand via various morphologically distinguishable progenitor cell types [16,17] and reach their final destination within the GZ before final maturation [18,19], with only relatively small numbers of mature neurons finally integrating into the existing neuronal networks [20,21]. The regulation of DG neurogenesis is mediated through multiple neurotrophic factors [22,23,24,25] and neurotransmitters [26,27,28], with a variety of intracellular signaling pathways involved [29,30]. Environmental factors, e.g., enriched environment and physical exercise, as important regulators of adult neurogenesis, have first been discovered in the DG [31,32,33]. On the functional level, enhanced DG neurogenesis is associated with an improved performance in hippocampus-dependent learning tasks, such as the Morris water maze [34]. 

aNSCs are not exclusively located in the PVR_V-SVZ_ and the DG, but also in more caudal PVRs of the midbrain around the caudal part of the 3rd down to the 4th ventricles (PVR_MB_), though in decreasing frequencies along the rostro-caudal axis [35,36]. Similarly to the aNSCs generated from PVR_S-SVZ_, stem or progenitor cells from more caudal PVRs are characterized in vitro and show similar properties as compared to PVR_V-SVZ_ aNSCs with the capacity of (in some cases limited) self-renewal and generation of tissue-specific differentiated cells, i.e., neurons and glial cells [9,35,36,37,38,39,40,41]. Indeed, aNSCs from the adult midbrain (tegmentum) show a regional commitment with a functional midbrain dopaminergic differentiation capacity in vitro [39]. Although the authors are aware of the ongoing discussion as to whether these proliferating cells are actually bona fide stem cells or restricted progenitor cells, we use the term aNSCs throughout this article to facilitate reading. Nevertheless, these stem or progenitor cells are either quiescent or very slowly proliferating in vivo, and active neurogenesis is not regularly observed in these brain regions [36,42,43]. To date, it remains enigmatic whether their quiescence is either due to cell-intrinsic or cell-extrinsic, i.e., local micro-environmental factors. 

Transplantation studies are one possibility to enlighten the interplay between cell-intrinsic and cell-extrinsic factors in the regulation of aNSC properties and have been carried out in a variety of transplantation sites with various graft origins: the homotopic transplantation of PVR_V-SVZ_ aNSCs provided large numbers of neurons with a typical olfactory bulb interneuron morphology [44], but other studies using heterotopic grafting of either PVR_V-SVZ_ aNSCs or neuroblasts yielded contradictory results, with either a predominant astroglial or neuronal differentiation independent of host specifications such as increased age or dopaminergic differentiation [45,46,47,48,49]. Similarly, hippocampal aNSCs display a robust neuronal differentiation after homo- and heterotopic grafting into neurogenic regions, but a strict astroglial differentiation otherwise [50,51,52,53]. A recent study even reported the acquisition of neuronal properties of grafted aNSCs through a fusion with resident neurons of the host brain [54]. Data on the properties of aNSCs from non-neurogenic regions are even more complex, since some studies report a neuronal differentiation of aNSCs derived from the *Substantia nigra* or spinal cord after grafting into neurogenic regions [55,56], though other studies even question the presence of bona fide aNSCs in these regions [36]. To our knowledge, transplantation studies of aNSCs from the caudal PVR_MB_ have not been carried out so far. The present study therefore aims at a deeper understanding of the properties of neural stem and progenitor cells from non-neurogenic regions after transplantation into a heterotopic neurogenic niche in vivo and at a thorough in vitro characterization of these cells to gain further knowledge on the capabilities of these stem cells as putative sources of new neurons in cell replacement therapies. In addition, the influence of physical exercise as a well-described environmental enhancer of adult neurogenesis on transplanted aNSCs has been assessed.

## 2. Material and Methods

### 2.1. Animals

Wild-type C57BL/6/J and C57BL/6-Tg(CAG-EGFP)131Osb/LeySopJ mice (both from Jackson Laboratories, US) were maintained under a 12-h light/dark cycle with constant temperature and humidity. C57BL/6-Tg(CAG-EGFP)131Osb/LeySopJ mice express an enhanced green fluorescent protein (EGFP) reporter under the control of a beta-actin promoter, leading to a widespread expression of GFP. Food and water were available ad libitum. All animal protocols were reviewed and approved by the Animal Welfare Committee at the Technische Universität Dresden and Landesdirektion Sachsen, Dresden, Germany (governmental authorities). Adult male C57BL/6/J mice were used as cell donors for in vitro experiments and transplant recipients, while transplants were derived from male C57BL/6-Tg(CAG-EGFP)131Osb/LeySopJ mice. 

### 2.2. Neural Stem Cell Culture and Differentiation

For the aNSC cultures, eight- to twelve-week-old male C57BL/6/J or C57BL/6-Tg(ACTB EGFP)1Osb/J mice, respectively, were killed by cervical dislocation, their brains removed and placed into a dissection solution (ice-cold Hank’s balanced salt solution supplemented with 1% penicillin/streptomycin and 2% glucose (all from Gibco, Tulsa, OK, USA)), as described earlier [36]. In brief, 500 µm coronal sections were microdissected for both cytological characterization and transplantation studies to obtain aNSCs derived from the V-SVZ (lateral wall of the lateral ventricles; PVR_V-SVZ_) and PVR of the midbrain (PVR_MB_)—due to a comparatively low aNSC yield in the caudal regions, we used aNSCs from the PVR of the caudal part of the 3rd ventricle adjusted to the midbrain nuclei down to the rostral part of the 4th ventricle. After incubation with both 0.1% trypsin (Sigma, St. Louis, MO, USA) and DNAse (40 mg/mL; Sigma, St. Louis, MO, USA), singularized cells were cultured in serum-free Neurobasal medium (+1% glutamate, 2% B27 supplement, 1% penicillin/streptomycin (all from Gibco, Carlsbad, CA, USA), 20 ng/mL epidermal growth factor (EGF), 20 ng/mL fibroblast growth factor 2 (FGF-2; both from Sigma, St. Louis, MO, USA) at 3%O_2_ in 6-well plates (TPP, Trasadingen, Switzerland) for 14 days. The media were changed once per week, and mitogens were added every two to three days. For the induction of terminal differentiation in vitro, neurospheres were plated on poly-d-lysine-coated cover slides in 24-well plates in Neurobasal medium (+100 μM dibuteryl(db)-cAMP (Sigma, St. Louis, MO, USA), 1% fetal calf serum (Gibco, Tulsa, OK, USA) and 10 ng/mL brain-derived neurotrophic factor (BDNF; Promega, Madison, WI, USA)) again at 3% atmospheric oxygen. The media were changed twice a week, and a differentiation was carried out for a total of 14 days. For immunocytochemistry, the cells were fixed with Accustain (Sigma, St. Louis, MO, USA) for 30 s. For transplantation studies, C57BL/6-Tg(ACTB EGFP)1Osb/J-derived aNSCs were expanded for 14 days exactly as described above and—immediately prior to grafting—singularized with trypsin and provided at 10^4^ cells/µL in a standard expansion medium. 

### 2.3. Immunocytochemistry

Immunocytochemistry was carried out using standard protocols [36] with the following primary antibodies: mouse anti-Nestin 1:500 (Merck Millipore, Burlington, MA, USA); rabbit anti-Olig2 1:500 (Merck-Millipore, Burlington, MA); rabbit anti-NG2 chondroitine sulfate 1:50 (Merck Millipore, Burlington, MA, USA); mouse anti-Tuj1 (βIII-tubulin) 1:500 (Covance, Princeton, NJ, USA); chicken anti-GFAP 1:1000 (Abcam, Cambridge, UK); rabbit anti-Ki67 1:500 (Abcam, Cambridge, UK). On the second day, the cells were incubated with the corresponding fluorescent-labeled secondary antibodies (1:500; all from Molecular Probes, Eugene, OR, USA; secondary antibodies’ fluorescence is indicated in the figure legends); cell nuclei were counterstained with 4,6-diamidino-2-phenylindole (DAPI). Images were captured using a fluorescence microscope (CXK41, Olympus, Shinjuku, Japan) at 20× magnification. 

### 2.4. Transplantation

Three days prior to transplantation, a subgroup of recipient mice was provided with running wheels until the end of the observation period, i.e., a total of 11 days (“runners”) for voluntary use. In compliance with the protocols of previous fundamental studies on the effects of voluntary wheel running on adult neurogenesis, mice were kept in groups of three per cage, and the functionality and usage of the running wheel was monitored by a revolution counter [31,57]. The individual running activity within the cage groups was not monitored, but the mentioned former fundamental studies on the neurogenic effects of voluntary wheel running showed little variability between individual mice [31,57]. For the transplantation procedures, expanded aNSCs were singularized and diluted to 10^4^ cells/µL. Male C57BL/6/J mice were anesthetized with isoflurane and placed in a rodent stereotaxic frame (Stoelting, Ireland) fitted with a mouse adaptor and stereotactically injected into the DG with 2 × 10^4^ cells at 0.1 µL/min using a precision injector (Nanoliter 2000; World Precision Instruments) at the following coordinates relative to bregma: ±1.6 mm mediolateral, −1.9 mm anterior–posterior and −1.9 mm dorsoventral, according to the stereotaxic coordinates provided by The Mouse Brain in Stereotaxic Coordinates by Franklin and Paxinos [58]. After transplantation, the needle was left in place for 10 min before retraction, and the incision sutured with absorbable filaments. For analgesia, the animals received oral tramadol (2.5 mg per 100 mL water) via their drinking water for 2 consecutive days.

For the labeling of proliferating cells, the animals were intraperitoneally injected with 50 mg/kg body weight 5′-bromo-2′-deoxy-uridine (BrdU; 10 mg/mL in 0.9% NaCl; Sigma, St. Louis, MO, USA) exactly every 24 h for 7 consecutive days. On day 7 after transplantation, the animals received a lethal overdose of ketamine/xylazine and were transcardially perfused with D-PBS followed by 4% paraformaldehyde (PFA; in D-PBS). The brains were carefully removed in toto, post-fixed in 4% PFA overnight, transferred to 30% sucrose for cryoprotection and stored at −80 °C for further analysis. The BrdU labeling, animal sacrifice, brain removal and post-fixation were all performed at the animal facility of the Max Planck Institute for Cell Biology and Genetics, in Dresden, Germany.

### 2.5. Immunohistochemistry

For immunohistochemistry, 40-µm-thick coronal sections were incubated with the following primary antibodies, according to standard protocols [59]: rat anti-BrdU 1:200, chicken anti-green fluorescent protein (GFP) 1:500, rabbit anti-Sox2 1:1000 (all from Abcam, Cambridge, UK), rabbit anti-NG2 chondroitine sulfate 1:100, mouse anti-neuronal nuclei (NeuN) 1:500 (both from Merck Millipore, Burlington, MA, USA). For detection of double and triple labeling, the following antibody combinations were used: anti-GFP/anti-BrdU/anti-Sox2; anti-GFP/anti-BrdU/NeuN; anti-GFP/anti-BrdU/anti-NeuroD1; anti-GFP/anti-BrdU/anti-NG2. The sections were then incubated with appropriate fluorochrome-conjugated secondary antibodies; cell nuclei were counterstained with Hoechst 33342. Fluorescence imaging was carried out on a confocal laser microscope (LSM 780; Carl Zeiss, Oberkochen, Germany) at 40× magnification. 

### 2.6. Quantifications and Statistical Analyses

For the quantification of grafted cells, we used quadruple immunohistochemical stainings with DAPI (cell nuclei), GFP (grafted cells), BrdU (proliferation) and a respective marker protein for the various cell types, as above. We chose every 6th coronal section of 40 µm thickness of the entire mouse brain (every 6th × 40 µm = 240 µm apart) and initially determined the correct graft position within the DG. Afterwards, we quantified the respective cell types in all sections containing grafted GFP^+^ cells. We did not predefine specific distances from the injection site for our analyses, but first identified the graft by GFP to allow the analysis of the complete grafts. The grafts were typically located at the tip of the injection tract, with the cell distribution adjacent to the injection tract suggestive of migratory movements toward neurogenic layers of neighboring host tissue. In all cases, the graft could be unequivocally identified, and the entire graft was analyzed by the counting procedure of every 6th section through the total graft in all stainings. Then, GFP^+^ cell counts indicative of the total surviving graft size were analyzed in the various DG subregions in the serial sections: the granular zone (GZ), the subgranular zone (SGZ) and the hilus. The data on total cell numbers and graft distribution comprised all three DG subregions, while for further detailed analyses of marker expression (cell type patterns), only transplanted cells located in the GZ and SGZ, the actual neurogenic niches, were included in the statistics. The initial quality inspections of the grafts included the detection of the vitality and localization of the grafts within the DG. Grafts with no vital cells (necrosis was detected by cell nuclei pyknosis in GFP/DAPI stainings; total of *n* = 3) and grafts outside the DG (total *n* = 3) were excluded from further analyses (see the Results section for details). The BrdU labeling index was herein defined as the proportion of cells of a given proliferative cell type with BrdU labeling after 7 days of consecutive BrdU application. For statistical comparisons by paired or unpaired two-sided *t*-tests or two-way mixed ANOVA, as appropriate (see the Results section for details), the SPSS software version 25.0 (SPSS, Chicago, IL, USA) was used. Post-hoc analyses were adjusted for α inflation using Bonferroni correction. *p*-values < 0.05 were considered significant. All data are expressed as mean ± SEM. 

## 3. Results

### 3.1. Comparative Characterization of Transplants Comprising aNSCs from PVR_V-SVZ_ and from PVR_MB_

**Figure 1:**
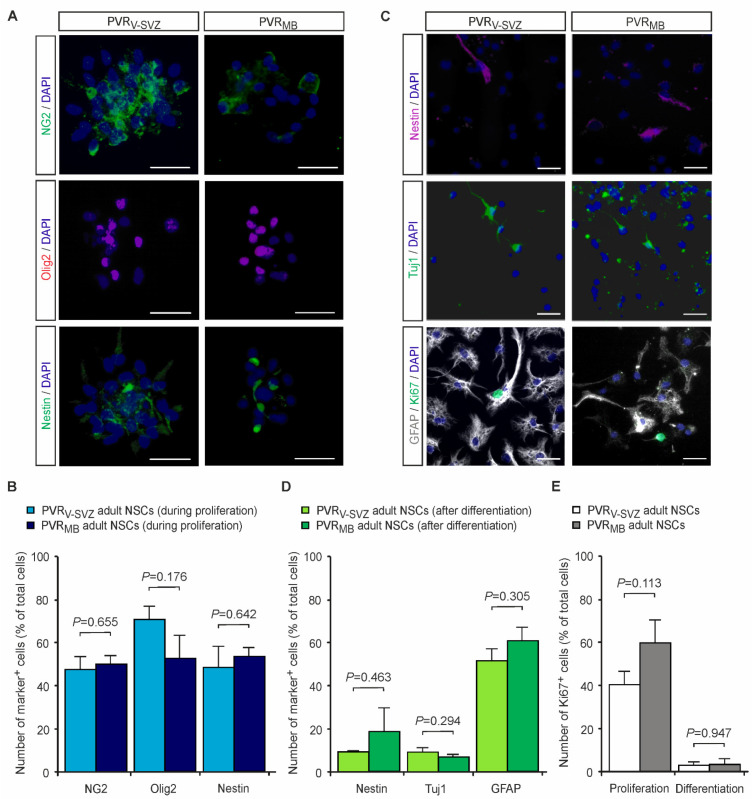
In vitro characterization of the transplants comprising aNSCs from PVR_V-SVZ_ or from PVR_MB_. (**A**,**B**) aNSCs from PVR_V-SVZ_ and PVR_MB_ were expanded for 14 days and analyzed by immunocytochemistry. (**A**) Representative fluorescence images of NG2 (green, upper panel), Olig2 (magenta, middle panel) and Nestin (green, lower panel) of aNSCs derived from PVR_V-SVZ_ (left column) and PVR_MB_ (right column) are shown. (**B**) Both aNSC types retained an undifferentiated progenitor cell state with a high expression of Nestin and oligodendroglial progenitor cell markers (NG2, Olig2) with no significant differences between aNSCs derived from the PVR_V-SVZ_ and PVR_MB_. (**C**,**D**) After the terminal differentiation of aNSCs for 14 days, we found small amounts of undifferentiated aNSCs and an overall astroglial commitment with a majority of GFAP^+^ astrocytes and smaller numbers of Tuj1^+^ neurons. Representative fluorescence images of Nestin (magenta, upper panel), tubulin III (Tuj1, green, middle panel) and GFAP (white, lower panel) of terminally differentiated aNSCs cultures derived from PVR_V-SVZ_ (right column) and PVR_MB_ (left column). (**D**) The quantification of undifferentiated Nestin^+^ cells as well as the astroglial and neuronal differentiation capacities revealed no significant differences between aNSCs derived from the PVR_V-SVZ_ and PVR_MB_. (**E**) Quantification of aNSCs expressing the proliferation marker Ki67 during proliferation and after differentiation (representative Ki67 stainings in (**C**), lower panel; the data during proliferation are from [9,39] using identical culture conditions as compared to the present study). We did not detect differences in the proliferation capacity between the two aNSC types in both culture conditions. Cell nuclei are counterstained with DAPI. Scale bars, 50 µm. All *p*-values are from unpaired two-sided *t*-tests (*n* = 3–7). Abbreviations: PVR_V-SVZ_—periventricular region of the ventricular-subventricular zone of the lateral ventricles; PVR_MB_—periventricular region of the mdbrain; aNSCs—adult neural stem cells; NG2—neural/glial antigen 2; Olig2—oligodendrocyte transcription factor 2; Tuj1—class III β-tubulin; GFAP—glial fibrillary acidic protein; DAPI—4′,6-diamidino-2-phenylindole.

Since the aNSCs from adult mouse PVRs are already characterized in vitro [9,35,36,37,38,39,40,41], we here comparatively characterize the transplants comprising aNSCs generated from the two periventricular germinal niches, namely the PVR_V-SVZ_ and the more caudal PVR_MB_, merely concerning their essential aNSC properties during expansion and after differentiation in vitro [36]. As depicted in Figure 1, PVR_MB_ aNSCs display a similar marker expression profile after two weeks of in vitro expansion in a non-adherent neurosphere cell culture system as compared to PVR_V-SVZ_ aNSCs. Approximately half of the cultured cells retained an early progenitor or stem cell state as indicated by the Nestin expression. In addition, high quantities of Olig2^+^ and NG2^+^ oligodendroglial-restricted progenitor cells developed from both aNSCs independent of their rostro-caudal origin along the ventricular neuraxis (Figure 1A,B). After the induction of terminal differentiation, both aNSC subtypes demonstrated a significant reduction of the expression of the NSC marker Nestin (*p* < 0.05 for both aNSC types, unpaired two-sided *t*-test; *n* = 3) with similar Nestin^+^ cell numbers in both aNSC types (Figure 1). Furthermore, we detected an overall astroglial differentiation with GFAP expression and the typical spider’s-web-like cell shape, but also robustly generated Tuj1^+^ cells with a typical neuronal morphology (Figure 1C,D). GalC^+^ oligodendrocytes were extremely rare in both forebrain- and midbrain-derived cultures (<0.1%; data not shown).

In addition, we used Ki67 to identify proliferating cells after 14 days of differentiation and compared these results with our previous data on aNSCs during proliferation using identical culture conditions as compared to the conditions used in the present study [9,39]. Ki67 is detected in the nucleus of proliferating cells in all active phases of the cell cycle, from the late G1 phase through the M phase, but is absent in non-proliferating and early G1 phase cells and in cells undergoing DNA repair [60,61,62]. As shown in Figure 1E, both aNSC types contain a similar percentage of proliferating cells during in vitro expansion in the non-adherent neurosphere cell culture system. In agreement with the stem cell marker expression, as described above, approximately half of the cultured cells expressed Ki67 independent of their brain region of origin. Fourteen days after the induction of terminal differentiation, both aNSC types demonstrated a marked reduction of proliferative cell numbers (*p* < 0.05 for both aNSC types) with similar amounts in both aNSC types (Figure 1C,E).

### 3.2. Grafts from PVR_MB_ Compared to PVR_V-SVZ_ Show Decreased Transplant Survival but Similar Proliferative Capacity

**Figure 2:**
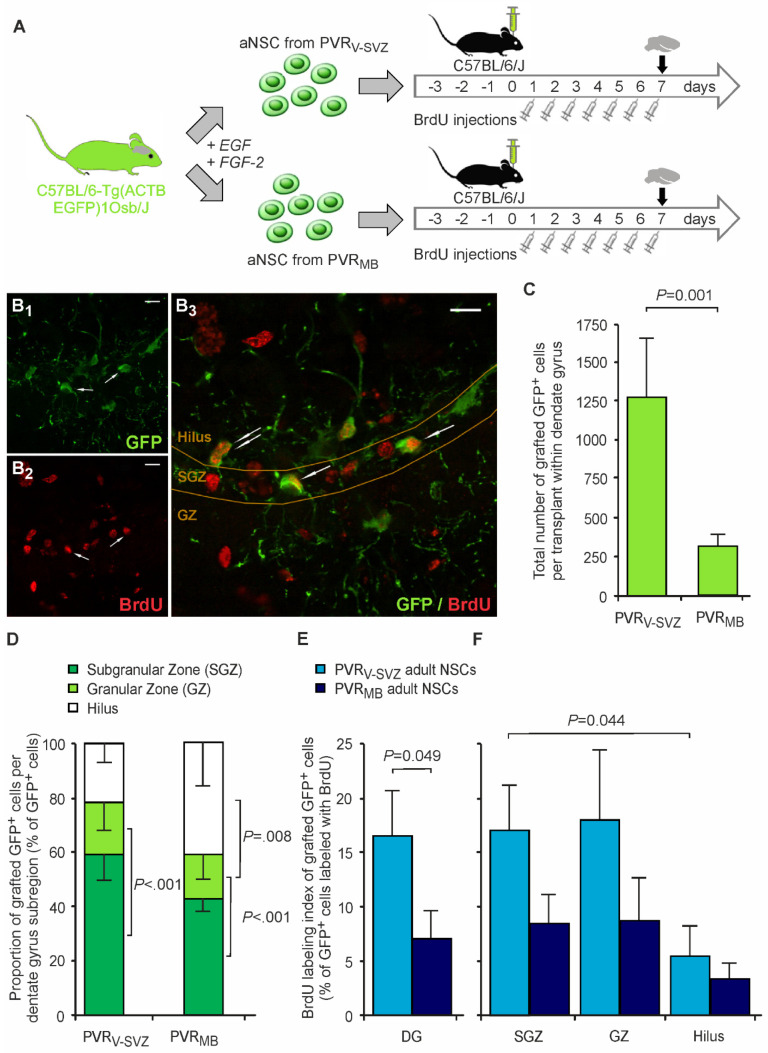
Graft survival and proliferation. (**A**) Schematic representation of transplantation experiments. C57BL/6-Tg(CAG-EGFP)131Osb/LeySopJ mice aged 8 to 12 weeks were sacrificed, the neurogenic regions (PVR_V-SVZ_, PVR_MB_) were dissected, and aNSCs expanded as neurospheres for 14 days. Afterwards, singularized aNSCs were transplanted into the hippocampal neurogenic niche (dentate gyrus) of wild-type mice. Both animal groups received intraperitoneal BrdU injections for 7 consecutive days after transplantation. Mice were sacrificed on day 7. (**B**) A representative fluorescence image of PVR_V-SVZ_ aNSC grafts correctly located within the DG shows that GFP^+^ cells (green, **B_1_**) are located in the SGZ and its adjacent regions. Daily BrdU injections labeled actively proliferating grafted cells (red, **B_2_**). Overview of the different subregions in the hippocampus, namely the granular zone, subgranular zone and hilus. Proliferating graft cells can be identified as GFP^+^/BrdU^+^ labeling (arrows; **B_3_**). Scale bars, 10 µm. (**C**) Quantitative immunohistochemistry revealed a low graft survival in both grafts, but significantly more GFP^+^-grafted cells in PVR_V-SVZ_ as compared to PVR_MB_ aNSC grafts one week post transplantation. (**D**) Subregional analyses of transplant location within the DG showed lower GFP^+^ cell numbers in GZ as compared to SGZ and hilus, with no differences between the two transplant origins (two-way mixed ANOVA with hippocampal region [SGZ, GS, hilus] and transplanted aNSC type [PVR_V-SVZ_ vs. PVR_MB_] as independent variables; see Supplementary Table S1 for the statistical results). (**E**,**F**) The daily BrdU labeling of proliferating cells revealed a higher proliferation capacity of grafted aNSCs derived from PVR_V-SVZ_ as compared to that of PVR_MB_ aNSCs (**E**). The absolute GFP^+^/BrdU^+^ cell counts within DG were 24.9 ± 9.9 cells per DG in PVR_V-SVZ_ grafts versus 4.2 ± 9.9 cells per DG in PVR_MB_ grafts (*p* = 0.249). (**F**) Subregional analyses of the transplant location displayed no relevant differences between the DG subregions with a tendency to lower proliferation rates in the hilus (two-way mixed ANOVA with hippocampal region [SGZ, GZ, hilus] and transplanted aNSC type [PVR_V-SVZ_ vs. PVR_MB_] as independent variables; see Supplementary Table S2 for the statistical results). *p*-values are from an unpaired two-sided *t*-test (**C**,**E**) or post-hoc *t*-tests with Bonferroni adjustment ((**D**,**F**); PVR_V-SVZ_ *n* = 8; PVR_MB_ *n* = 22). Abbreviations: EGF—epidermal growth factor; FGF-2—fibroblast growth factor-2; PVR_V-SVZ_—periventricular region of the ventricular-subventricular zone of the lateral wall of the lateral ventricles; PVR_MB_—periventricular region of the midbrain; aNSCs—adult neural stem cells; BrdU—5′-bromo-2′-desoxyuridine; DG—dentate gyrus of the hippocampus; SGZ—subgranular zone; GZ—granular zone; GFP—green fluorescent protein.

We used GFP expression to identify the grafted aNSCs and their progenies in contrast to resident host aNSCs (see Figure 2A for the experimental design). Daily BrdU labeling during the first 7 days post grafting allowed for a thorough assessment of transplant proliferation and a concomitant differentiation within the host microenvironment in the DG. On day 7, recipient mice were sacrificed, and the transplant sizes analyzed. We included only transplants with correct localization within the DG in the analyses (*n* = 13 for PVR_V-SVZ_ and *n* = 24 for PVR_MB_ transplants, respectively). The PVR_V-SVZ_-transplanted group consisted of nine regular animals and four animals provided with running wheels (“runners”). In quality inspections of the transplants, one PVR_V-SVZ_ (8%) and two PVR_MB_ transplants (8%) were completely necrotic, with no viable cells, most likely due to the implantation procedure (*p* = 1.000, Fisher’s Exact test). These transplants were not included in the analyses.

On average, ~1250 GFP^+^-grafted cells (~6.3% of transplanted cells) were detected in PVR_V-SVZ_-aNSC-transplanted mice, while cell counts were more than 4.1 times lower for PVR_MB_ grafts (Figure 2B,C). Although we did not include analyses of cell death or the apoptosis of grafted cells in our protocol, our approach to analyze the entire graft regardless of its exact localization (see the Material & Methods section), with low cell counts in both grafts, indicates a limited graft survival. Since resident hippocampal aNSCs are strictly located in the SGZ of the DG and their progeny migrates only into the adjacent GZ, the distribution and properties of grafted cells within the various DG subregions is also of interest when analyzing graft survival and fate. Since the transplantation needle tract is perpendicular to the DG formation, the accuracy of the transplantation approach does not allow for the exact placement of the grafts within DG subregions, placing them instead within the whole DG, including the hilus. However, GFP^+^-grafted aNSCs preferentially aligned in the genuine neurogenic niche of the SGZ as compared to the GZ, with no major differences between the two transplant origins (Figure 2D). A relevant proportion of GFP^+^ cells was also detected in the hilus in both grafts (Figure 2B,D). The BrdU-labeling of proliferating cells revealed a higher proliferation rate of grafted aNSCs derived from PVR_V-SVZ_ compared to that of PVR_MB_ aNSCS within the DG, with similar patterns in DG subregions though not reaching significant differences between the grafts (Figure 2E,F). 

### 3.3. Grafted aNSCs from both PVR_V-SVZ_ and PVR_MB_ Show Similar Neural Stem Cell Properties

**Figure 3:**
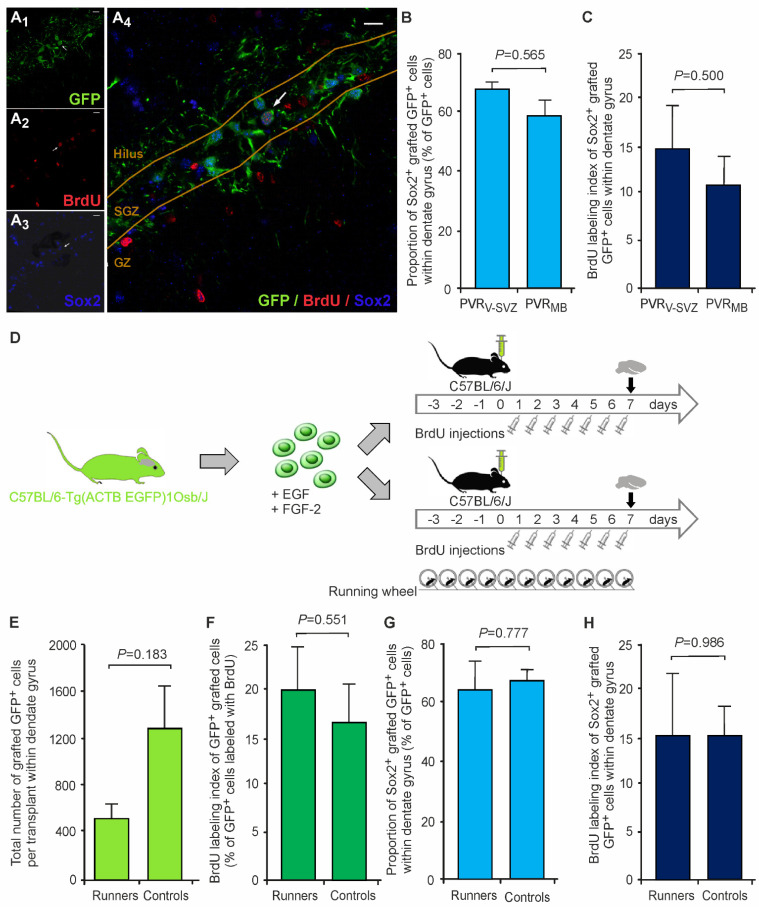
Sox2^+^ neural stem cell properties of grafted aNSCs in the host under control and physical exercise conditions. (**A**) Representative triple fluorescence immunostaining of a PVR_V-SVZ_ graft correctly located within the DG (the arrow illustrates a GFP^+^/BrdU^+^/Sox2^+^-grafted aNSC; **A_4_**). GFP identifies transplanted cells in contrast to resident cells (green, **A_1_**), while BrdU (red, **A_2_**) indicates cells which actively proliferate after transplantation. Sox2 serves as a marker for stem and progenitor cells (blue, **A_3_**). Scale bars, 10 µm. (**B**) Relative Sox2^+^ cell counts with the DG normalized to total GFP^+^-grafted cell counts did not differ between the two grafts (PVR_V-SVZ_ vs. PVR_MB_). (**C**) Likewise, the relative amounts of proliferating BrdU^+^ cells among the persistent aNSCs (GFP^+^/Sox2^+^/BrdU^+^ cells) were similar in both grafts. The *p*-values are from unpaired two-sided *t*-tests (PVR_V-SVZ_ *n* = 6; PVR_MB_ *n* = 21). (**D**–**H**) Effects of physical exercise (“runners”) on survival, proliferation and stemness of grafted PVR_V-SVZ_ aNSCs within the DG. (**D**) Schematic representation of transplantation experiments. aNSCs from PVR_V-SVZ_ were transplanted into the hippocampal neurogenic niche (dentate gyrus) of wild-type mice, which were divided into two groups: animals in standard cages and “runners” provided with a running wheel during days −3 to 7 post transplantation. Both animal groups received intraperitoneal BrdU injections for 7 consecutive days after transplantation. Mice were sacrificed on day 7. In general, significantly more GFP^+^-grafted cells in PVR_V-SVZ_ as compared to PVR_MB_ aNSC grafts one week post transplantation. Proliferation and stemness of grafted PVR_V-SVZ_ aNSCs within the DG. There were no differences either in graft survival (**E**), the relative amounts of GFP^+^/BrdU^+^-proliferating cells between runners and controls (**F**), the relative amounts of total Sox2^+^ cells (**G**) or their proliferative BrdU^+^ subpopulation (**H**). The *p*-values are from unpaired two-sided *t*-tests (runners *n* = 4; controls *n* = 7). Abbreviations: PVR_V-SVZ_—periventricular region of the ventricular-subventricular zone of the lateral ventricles; PVR_MB_—periventricular region of the midbrain; aNSC—adult neural stem cell; SGZ—subgranular zone; GZ—granular zone; GFP—green fluorescent protein; BrdU—5′-bromo-2′-desoxyuridine.

Since the aNSCs from non-neurogenic regions are either quiescent in vivo or proliferate very slowly [36], their actual neurogenic differentiation potential in vivo has not been sufficiently determined so far. We therefore comparatively assessed the stemness and proliferation capacities of the two grafts within the DG 7 days after transplantation (Figure 3A,B). With around 2/3 of grafted cells retaining their stem cell status in both PVR_V-SVZ_ and PVR_MB_ aNSC grafts, the differentiation progress one week after transplantation was only modest. The amounts of grafted Sox2^+^ cells within the DG did not show any differences between the grafted aNSC types (Figure 3B). The BrdU labeling index of Sox2^+^ cells showed that ~12–16% of all Sox2^+^-grafted cells found proliferated after transplantation with—interestingly and in contrast to the entire aNSC graft (Figure 2E,F)—no differences between the two grafts (Figure 3C).

When analyzing the subregional distribution patterns of grafted cells within the main neurogenic regions (SGZ and GZ), we did not detect any differences of relative GFP^+^/Sox2^+^ aNSC counts between the DG subregions or between the grafted aNSC type (*p* > 0.05 for all comparisons by a two-way mixed ANOVA; Supplementary Figure S1A, Table S3). The BrdU labeling index of Sox2^+^ cells showed that ~10–29% of all Sox2^+^-grafted cells proliferated after transplantation regardless of both the DG subregion and the graft type (Supplementary Figure S1B, Table S4). 

### 3.4. Voluntary Wheel Running of Host Animals Does Not Change PVR_V-SVZ_ Graft Properties

Since the graft survival rates were quite low, a subgroup of PVR_V-SVZ_-transplanted animals was provided with running wheels within their regular home cages (“runners”) three days prior to transplantation to induce physical exercise (see Figure 3D for the scheme of the experimental design). This experimental paradigm using wheel running is a well-established environmental stimulator of resident neural stem cell proliferation and adult neurogenesis [22,31,33] and might therefore also exert a positive influence on grafted aNSCs, though—to our knowledge—this has not been studied so far. Although there were numerically more GFP^+^-grafted cells in controls compared to runners, these differences were not significant between running host mice and controls (Figure 3E). There were also no differences between runners and controls in the relative amounts of proliferating GFP^+^/BrdU^+^ cells (Figure 3F) or in the relative amounts of Sox2^+^ stem cells or their proliferative Sox2^+^/BrdU^+^ progenies (Figure 3G,H). Consistently, we did not observe significant differences between runners and controls in the DG subregional distribution patterns of these cell populations (*p* > 0.05 for all comparisons by a two-way mixed ANOVA; Supplementary Tables S5–S7). 

### 3.5. Grafted aNSCs from PVR_MB_ Are Not Capable of Early Neuronal Differentiation

**Figure 4:**
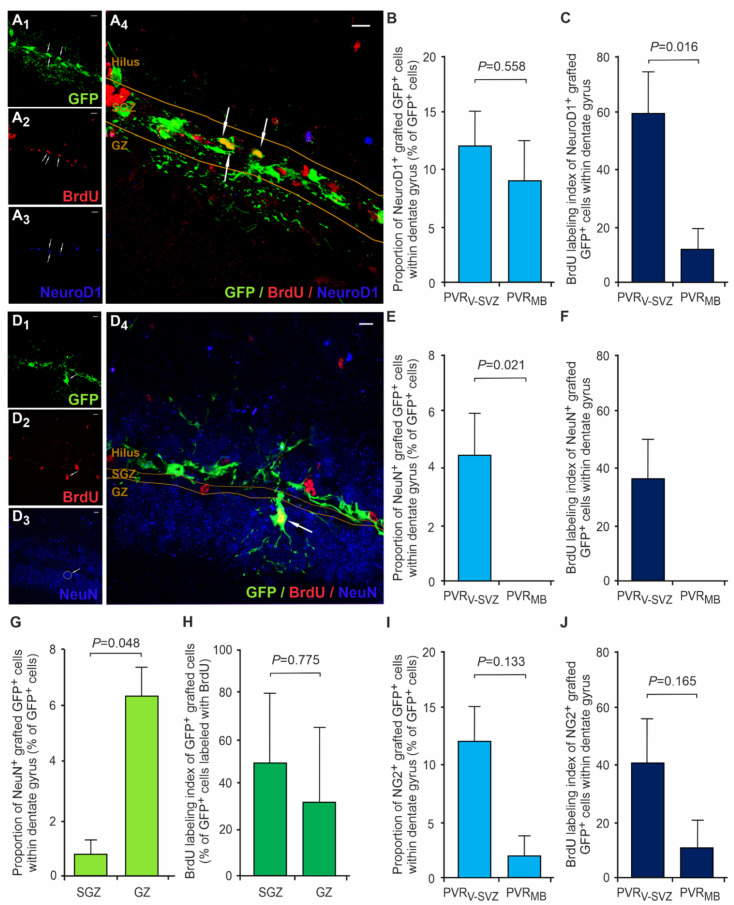
Differentiation capacity of grafted aNSCs depending on the graft origin. (**A**) Representative triple fluorescence immunostaining of a PVR_V-SVZ_ graft within the DG (the arrows illustrate a GFP^+^/BrdU^+^/NeuroD1^+^-grafted NSC; **A_4_**). GFP identifies the transplanted cells (green, **A_1_**), BrdU (red, **A_2_**) indicates the cells proliferating after transplantation, and NeuroD1 serves as a marker for immature neurons (blue, **A_3_**). Scale bars, 10 µm. (**B**,**C**) The relative NeuroD1^+^ cell counts with the DG normalized to total GFP^+^-grafted cell counts did not differ between the two grafts (PVR_V-SVZ_ vs. PVR_MB_). (**C**) The daily BrdU labeling of proliferating cells revealed a higher proliferation capacity of grafted NeuoD1^+^ cells derived from PVR_V-SVZ_ as compared to that of PVR_MB_ aNSCs. The *p*-values are from unpaired two-sided *t*-tests (PVR_V-SVZ_ *n* = 8; PVR_MB_ *n* = 7). (**D**) Representative triple fluorescence immunostaining of a PVR_V-SVZ_ graft within the DG (the arrows illustrate a GFP^+^/BrdU^+^/NeuN^+^-grafted cell; **D_4_**). GFP identifies the transplanted cells, (green, **D_1_**), BrdU (red, **D_2_**) indicates the cells which proliferated after transplantation, and NeuN serves as a marker for neurons (blue, **D_3_**). Scale bars, 10 µm. (**E**–**H**) The relative NeuN^+^ cell counts with the DG normalized to total GFP^+^-grafted cell counts demonstrated an exclusive neuronal differentiation in PVR_V-SVZ_ grafts. (**F**) The BrdU labeling index shows that ~37% of NeuN^+^ derived from previously proliferating cells. Notably, most grafted NeuN^+^ neurons are located within the GZ (**G**; arrow in **D_4_**), with no differences in the BrdU labeling index (**H**). The *p*-values are from unpaired (**E**,**F**) or paired (**G**,**H**) two-sided *t*-tests (PVR_V-SVZ_ *n* = 7; PVR_MB_ *n* = 8). (**I**,**J**) Relative NG2^+^ polydendrocyte counts with the DG normalized to total GFP^+^-grafted cell counts varied markedly and did not differ between the two grafts (PVR_V-SVZ_ vs. PVR_MB_). The relative amounts of BrdU^+^ polydendrocytes were similar in both grafts (J). The *p*-values are from unpaired two-sided *t*-tests (PVR_V-SVZ_ *n* = 6; PVR_MB_ *n* = 5). Abbreviations: PVR_V-SVZ_—periventricular region of the ventricular-subventricular zone of the lateral wall of the lateral wall of the lateral ventricles; PVR_MB_—periventricular region of the midbrain; aNSC—adult neural stem cell; SGZ—subgranular zone; GZ—granular zone; GFP—green fluorescent protein; BrdU—5′-bromo-2′-desoxyuridine; NeuroD1—neuronal differentiation 1 protein; NeuN—neuronal nuclei protein; DG—dentate gyrus.

The early neuronal lineage commitment of grafted cells was determined by NeuroD1 expression analyses (Figure 4A,B). Since the majority of grafted cells retained stem cell properties, the overall NeuroD1+ cell amounts were rather low in both transplant subtypes with 10–12% NeuroD1+ cells per total GFP+ cells (Figure 4B). The BrdU labeling index of NeuroD1+ cells showed a significant difference between graft types with ~60% proliferative cells of all NeuroD1+-grafted cells in PVRV-SVZ compared to ~12% in PVRMB grafts (Figure 4C). In contrast to the Sox2^+^ stem cell population, with its equal distribution between SGZ and GZ, the distribution of grafted NeuroD1^+^ cells within the DG displayed significantly more NeuroD1^+^ cells within SGZ as compared to GZ, with no differences between the grafts (*p* < 0.05; two-way mixed ANOVA with post-hoc *t*-test; Supplementary Figure S1C, Table S8). The BrdU labeling index of NeuroD1^+^ cells displayed a significant difference between the grafts in SGZ (Supplementary Figure S1D).

The terminal differentiation into mature NeuN^+^ neurons was a rare event, exclusively observed in PVR_V-SVZ_ grafts within the first 7 days post transplantation, with more grafted NeuN^+^ cells within the GZ as compared to the SGZ (Figure 4D–H). Approximately 37% of GFP^+^/NeuN^+^ cells were positive for the proliferation marker BrdU, and thus presumably derived from actively dividing the aNSCs after transplantation (Figure 4F). 

### 3.6. Grafted aNSCs from both PVR_V-SVZ_ and PVR_MB_ Show Limited Polydendrocyte Potential

Since astroglial instead of neuronal differentiation has often been observed in heterotopic transplantation studies [46,63], we assessed the appearance of oligodendrocyte progenitor cells, so-called polydendrocytes, within the grafts using the marker NG2 [64]. Interestingly, the NG2 counts varied pronouncedly and were comparatively low in our study, with <15% of NG2^+^-grafted cells irrespective of the DG subregion and the graft origin (Figure 4I, Supplementary Figure S2A,B, Table S9). The BrdU labeling index of GFP^+^/NG2^+^ within DG ranged from 46 to 66%, with no differences between the grafts (Figure 4J, Supplementary Figure S2A,C).

## 4. Discussion

The concept of a strict confinement of aNSCs to the two classical neurogenic regions of the PVR_V-SVZ_ of the lateral ventricles and the DG within the hippocampus [65,66] has been challenged for quite some time [43,55,67,68]. In previous studies, our group and others already demonstrated the presence of comparatively low numbers of quiescent neural stem and progenitor cells along the whole ventricular neuraxis in vivo [35,36], though spontaneous neurogenesis cannot be observed in these ’non-neurogenic’ regions [36,42]. This might be due, to a certain extent, to local inhibitory factors, such as norepinephrine innervation from *Locus coeruleus* [9]. Norepinephrine suppresses aNSC proliferation in vitro and promotes their cell cycle exit and subsequent neuronal differentiation through β-adrenoceptor signaling [9]. Therefore, it remains controversial whether aNSCs isolated from these non-neurogenic caudal PVRs actually possess a similar cell-intrinsic proliferation capacity and neurogenic potential as compared to the well-characterized aNSCs of the PVR_V-SVZ_ [43]. The present study was thus designed to compare the proliferation capacity and early neurogenic differentiation potential of aNSCs from PVR_V-SVZ_ and from caudal PVR_MB_ after transplantation into the pro-neurogenic microenvironment of the DG.

The initial in vitro characterization of the transplants demonstrates that aNSCs from the caudel PVR_MB_ do actually possess a similar neurogenic potential in vitro compared to aNSCs derived from the PVR_V-SVZ_, the neurogenic niche with the highest proliferative activity in vivo [36,66]. In contrast to the in vivo situation, culture conditions lead to a robust neurosphere formation [36] and the preservation of an undifferentiated proliferative state with a retention of stem and progenitor cell markers such as Nestin, Olig2 and NG2—not only in PVR_V-SVZ_ aNSCs, but also in PVR_MB_ aNSCs. After the induction of terminal differentiation, both aNSC subtypes robustly generated new neurons in vitro, though the majority of cells differentiated into astrocytes, most likely due to our differentiation protocol, which was not specifically designed to enforce neuronal differentiation [39]. However, the present study was not designed to distinguish between neural progenitor cells and bona fide stem cells, especially concerning the indefinite self-renewal capacity of the latter cell type. Indeed, this has already been questioned for proliferating aNSCs from outside the neurogenic regions of V-SVZ and DG [35], but also for different regions within the PVR_V-SVZ_ [69]. Nevertheless, PVR_MB_ aNSCs are capable of generating new neurons in a permissive environment in vitro, which leads to the question of whether such a pro-neurogenic microenvironment in vivo would also allow for active proliferation and neuronal differentiation.

After the transplantation into the DG as a pro-neurogenic environment, aNSCs from both the neurogenic niche PVR_V-SVZ_ and the non-neurogenic region PVR_MB_ were able to survive and proliferate, as indicated by the BrdU uptake. In our study, the overall fractions of surviving GFP^+^-grafted cells 7 days post transplantation were quite low in both graft subtypes with 4-times lower GFP^+^ cell counts of PVR_MB_ aNSCs. The reasons for this striking difference remain enigmatic, but might include the higher proliferation capacity of PVR_V-SVZ_ aNSCs after transplantation as compared to PVR_MB_ aNSCs. In addition, a higher susceptibility of PVR_MB_ aNSCs to the implantation process, including mechanical stress or enzymatic singularization leading to a lower cell survival of PVR_MB_ aNSCs, might also contribute to the differences in cell counts between the two graft types. Nevertheless, these survival rates of PVR_V-SVZ_ aNSCs are in line with other reports on short-period transplantations with various NSC preparations [54,63,70,71]. In contrast, long-term studies, up to several months after transplantation, report considerably higher numbers of remaining grafted cells [49]. These generally low cell counts can be attributed to the transplantation process itself, leading to a disruption of the local cytoarchitecture, with a disturbed vasculature, a reduced blood oxygen supply, microglia transformation and the release of toxic and proinflammatory cytokines [72,73,74]. 

Physical exercise, such as wheel-running, is a well-described positive regulator of genuine hippocampal neurogenesis, particularly by enhancing neural stem cell proliferation [22,31,33], but it is currently unclear whether non-hippocampal aNSCs also benefit from this sort of environmental manipulation. Although we used an experimental paradigm known to increase the proliferation of genuine Sox2^+^ hippocampal stem cells in adult mice [28], we observed that physical exercise did not alter either the proliferation and survival of PVR_V-SVZ_ grafts in the DG or the proportion of proliferating Sox2^+^ stem cells, suggesting that PVR-derived aNSCs are not able to receive the signals mediating exercise-induced effects on hippocampal neurogenesis. This fact might be related to differences in cell-intrinsic properties between PVR-derived aNSCs and hippocampal stem cells, such as their sensitivity to neurotransmitters regulating aNSC proliferation [9]. Since neurotransmitters and other humoral factors [10,22,23,24,26,27,28], as pivotal regulators of adult neurogenesis, presumably need a sufficient integration of the transplanted aNSCs into the hippocampal circuitries, the limited tissue integration of the grafts after a period of 7 days might also restrict the sensitivity of the grafted aNSCs to exercise-induced cues. Moreover, the disturbed microarchitecture due to the transplantation procedure itself needs to be considered, since it might also alter the molecular effector pathways influenced by physical exercise. Since the present studies were primarily designed to comparatively investigate PVR aNSC grafts from different regions, and physical exercise was tested to increase survival and the proliferation of grafted cells, future studies should further characterize the factors responsible for the lack of effects of exercise on PVR-derived aNSCs within the hippocampal neurogenic niche. Long-term observation studies of transplants to allow the grafted cells to integrate into the hippocampal formation are recommended. 

Although both types of PVR-derived grafts survived and proliferated actively in this unfamiliar microenvironment, the neuronal differentiation with detection of NeuN^+^ young neurons was exclusively confined to PVR_V-SVZ_ aNSCs grafts within the first week post grafting. Interestingly, grafted PVR_V-SVZ_ aNSCs displayed the physiological migration process during the differentiation from a higher proportion of NeuroD1^+^ cells within the SGZ to more NeuN^+^ neurons within the GZ, indicating that the grafted PVR_V-SVZ_ aNSCs are able to percept signaling cues from the host DG, facilitating either an accelerated neuronal differentiation or a directed migration process. Since the present study was only designed to determine aNSC properties and early neuronal differentiation steps, it cannot clarify whether the lack of neurons in PVR_MB_ grafts is actually due to a reduced intrinsic differentiation potential per se or to a slower differentiation propensity in the more quiescent PVR_MB_ aNSC population, not completely depicted by the observation period in the present study. On the one hand, our in vitro data, showing similar neuronal differentiation capacities of both aNSC types after 14 days, imply a slower differentiation behavior of PVR_MB_ compared to PVR_V-SVZ_ grafts. On the other hand, the lack of terminal neuronal differentiation in PVR_MB_ grafts, together with similar numbers of NeuroD1^+^ immature neurons but a higher BrdU proliferation index of NeuroD1^+^ cells in PVR_V-SVZ_ grafts, suggest that PVR_MB_ grafts display a limited proliferation capacity of neuronal progenitors as well as a limited terminal neuronal differentiation capacity within the hippocampal neurogenic niche. A fusion of grafted fetal NSC lines with host neuronal elements has been recently described [54], similar to the phenomenon observed several years ago with bone marrow-derived cells [75]. This fusion process of NSCs seems to be specific of host neurons and is not observed with other brain cell types. It only involves approx. 3–8% of grafted cells [54]. Although there are no data on aNSCs on cell fusion after transplantation, this phenomenon might have partly influenced our results on early neuronal differentiation, but most likely not our data on NSC properties and their proliferation capacities. Long-term graft observation experiments combined with BrdU birthdating and a double labeling of both host and grafted cells to securely exclude cell fusion are needed to further characterize the differences between aNSCs derived from various PVRs throughout the ventricular system. 

## 5. Conclusions

Although midbrain aNSCs show a similar proliferation and neurogenic differentiation capacity in comparison to highly active PVR_V-SVZ_ aNSCs in vitro, our study provides further evidence that aNSCs from caudal PVR_MB_ as a ‘non-neurogenic region’ actually exhibit different intrinsic properties, with a reduced response to neurogenic stimuli after transplantation into a permissive microenvironment in vivo. The major differences between the two aNSC types are a markedly inferior survival and proliferation potential as well as a reduced neuronal differentiation capacity in aNSCs from caudal PVR_MB_ as compared to PVR_V-SVZ_ aNSCs. Although these differences might be—in part—related to the transplantation procedure and the short-term study design, as discussed in detail above, our data strongly imply important cell-intrinsic differences between aNSCs from neurogenic compared to non-neurogenic PVRs with respect to their neurogenic potential and/or their sensitivity to neurogenic cues. Future experiments investigating long-term graft survival are warranted to confirm our data. These future studies might be combined with experiments on the influence of parallel local brain damage as a factor influencing aNSC fate [76] on the different cellular engraftment of the two aNSC subtypes. These results are of special interest for the understanding of the potential limitations of novel cell replacement therapies, e.g., in certain midbrain disorders such as Parkinson´s disease.

## Data Availability

The data used to support the findings of this study are available from the corresponding author upon request.

## Supplementary Materials

The following are available online at https://www.mdpi.com/article/10.3390/cells10113021/s1, Tables S1–S9: Statistical results of two-way mixed ANOVA, Figure S1: Subregional distribution of Sox2+ neural stem cells and NeuroD1+ immature neurons 7 days after transplantation, Figure S2: Subregional distribution of NG2+ oligodendroglial progenitor cells 7 days after transplantation.

## References

[1] Lim D.A., Alvarez-Buylla A. (2016). The Adult Ventricular-Subventricular Zone (V-SVZ) and Olfactory Bulb (OB) Neurogenesis. Cold Spring Harb. Perspect. Biol..

[2] Zigova T., Pencea V., Wiegand S.J., Luskin M.B. (1998). Intraventricular administration of BDNF increases the number of newly generated neurons in the adult olfactory bulb. Mol. Cell. Neurosci..

[3] Delgado A.C., Ferron S.R., Vicente D., Porlan E., Perez-Villalba A., Trujillo C.M., D’Ocon P., Farinas I. (2014). Endothelial NT-3 delivered by vasculature and CSF promotes quiescence of subependymal neural stem cells through nitric oxide induction. Neuron.

[4] Ahn S., Joyner A.L. (2005). In vivo analysis of quiescent adult neural stem cells responding to Sonic hedgehog. Nature.

[5] Lim D.A., Tramontin A.D., Trevejo J.M., Herrera D.G., Garcia-Verdugo J.M., Alvarez-Buylla A. (2000). Noggin antagonizes BMP signaling to create a niche for adult neurogenesis. Neuron.

[6] Shimogori T., VanSant J., Paik E., Grove E.A. (2004). Members of the Wnt, Fz, and Frp gene families expressed in postnatal mouse cerebral cortex. J. Comp. Neurol..

[7] Brezun J.M., Daszuta A. (1999). Depletion in serotonin decreases neurogenesis in the dentate gyrus and the subventricular zone of adult rats. Neuroscience.

[8] Hoglinger G.U., Rizk P., Muriel M.P., Duyckaerts C., Oertel W.H., Caille I., Hirsch E.C. (2004). Dopamine depletion impairs precursor cell proliferation in Parkinson disease. Nat. Neurosci..

[9] Weselek G., Keiner S., Fauser M., Wagenfuhr L., Muller J., Kaltschmidt B., Brandt M.D., Gerlach M., Redecker C., Hermann A. (2020). Norepinephrine is a negative regulator of the adult periventricular neural stem cell niche. Stem Cells.

[10] Merkle F.T., Mirzadeh Z., Alvarez-Buylla A. (2007). Mosaic organization of neural stem cells in the adult brain. Science.

[11] Fuentealba L.C., Rompani S.B., Parraguez J.I., Obernier K., Romero R., Cepko C.L., Alvarez-Buylla A. (2015). Embryonic Origin of Postnatal Neural Stem Cells. Cell.

[12] Ninkovic J., Steiner-Mezzadri A., Jawerka M., Akinci U., Masserdotti G., Petricca S., Fischer J., von Holst A., Beckers J., Lie C.D. (2013). The BAF complex interacts with Pax6 in adult neural progenitors to establish a neurogenic cross-regulatory transcriptional network. Cell Stem Cell.

[13] Zhou Q., Dalgard C.L., Wynder C., Doughty M.L. (2011). Histone deacetylase inhibitors SAHA and sodium butyrate block G1-to-S cell cycle progression in neurosphere formation by adult subventricular cells. BMC Neurosci..

[14] Wu H., Coskun V., Tao J., Xie W., Ge W., Yoshikawa K., Li E., Zhang Y., Sun Y.E. (2010). Dnmt3a-dependent nonpromoter DNA methylation facilitates transcription of neurogenic genes. Science.

[15] Kempermann G., Song H., Gage F.H. (2015). Neurogenesis in the Adult Hippocampus. Cold Spring Harb. Perspect. Biol..

[16] Filippov V., Kronenberg G., Pivneva T., Reuter K., Steiner B., Wang L.P., Yamaguchi M., Kettenmann H., Kempermann G. (2003). Subpopulation of nestin-expressing progenitor cells in the adult murine hippocampus shows electrophysiological and morphological characteristics of astrocytes. Mol. Cell. Neurosci..

[17] Seri B., Garcia-Verdugo J.M., McEwen B.S., Alvarez-Buylla A. (2001). Astrocytes give rise to new neurons in the adult mammalian hippocampus. J. Neurosci..

[18] Fukuda S., Kato F., Tozuka Y., Yamaguchi M., Miyamoto Y., Hisatsune T. (2003). Two distinct subpopulations of nestin-positive cells in adult mouse dentate gyrus. J. Neurosci..

[19] Steiner B., Klempin F., Wang L., Kott M., Kettenmann H., Kempermann G. (2006). Type-2 cells as link between glial and neuronal lineage in adult hippocampal neurogenesis. Glia.

[20] Brandt M.D., Jessberger S., Steiner B., Kronenberg G., Reuter K., Bick-Sander A., von der Behrens W., Kempermann G. (2003). Transient calretinin expression defines early postmitotic step of neuronal differentiation in adult hippocampal neurogenesis of mice. Mol. Cell. Neurosci..

[21] Kempermann G., Gast D., Kronenberg G., Yamaguchi M., Gage F.H. (2003). Early determination and long-term persistence of adult-generated new neurons in the hippocampus of mice. Development.

[22] Farmer J., Zhao X., van Praag H., Wodtke K., Gage F.H., Christie B.R. (2004). Effects of voluntary exercise on synaptic plasticity and gene expression in the dentate gyrus of adult male Sprague-Dawley rats in vivo. Neuroscience.

[23] Neeper S.A., Gomez-Pinilla F., Choi J., Cotman C.W. (1996). Physical activity increases mRNA for brain-derived neurotrophic factor and nerve growth factor in rat brain. Brain Res..

[24] Fabel K., Fabel K., Tam B., Kaufer D., Baiker A., Simmons N., Kuo C.J., Palmer T.D. (2003). VEGF is necessary for exercise-induced adult hippocampal neurogenesis. Eur. J. Neurosci..

[25] Carro E., Nuñez A., Busiguina S., Torres-Aleman I. (2000). Circulating insulin-like growth factor I mediates effects of exercise on the brain. J. Neurosci..

[26] Song J., Zhong C., Bonaguidi M.A., Sun G.J., Hsu D., Gu Y., Meletis K., Huang Z.J., Ge S., Enikolopov G. (2012). Neuronal circuitry mechanism regulating adult quiescent neural stem-cell fate decision. Nature.

[27] Esposito M.S., Piatti V.C., Laplagne D.A., Morgenstern N.A., Ferrari C.C., Pitossi F.J., Schinder A.F. (2005). Neuronal differentiation in the adult hippocampus recapitulates embryonic development. J. Neurosci..

[28] Klempin F., Beis D., Mosienko V., Kempermann G., Bader M., Alenina N. (2013). Serotonin is required for exercise-induced adult hippocampal neurogenesis. J. Neurosci..

[29] Munoz M.D., Antolin-Vallespin M., Tapia-Gonzalez S., Sanchez-Capelo A. (2016). Smad3 deficiency inhibits dentate gyrus LTP by enhancing GABAA neurotransmission. J. Neurochem..

[30] Zhang Z., Gao F., Kang X., Li J., Zhang L., Dong W., Jin Z., Li F., Gao N., Cai X. (2015). Exploring the potential relationship between Notch pathway genes expression and their promoter methylation in mice hippocampal neurogenesis. Brain Res. Bull..

[31] van Praag H., Kempermann G., Gage F.H. (1999). Running increases cell proliferation and neurogenesis in the adult mouse dentate gyrus. Nat. Neurosci..

[32] Fabel K., Wolf S.A., Ehninger D., Babu H., Leal-Galicia P., Kempermann G. (2009). Additive effects of physical exercise and environmental enrichment on adult hippocampal neurogenesis in mice. Front. Neurosci..

[33] Kobilo T., Liu Q.R., Gandhi K., Mughal M., Shaham Y., van Praag H. (2011). Running is the neurogenic and neurotrophic stimulus in environmental enrichment. Learn. Mem..

[34] Kempermann G., Kuhn H.G., Gage F.H. (1997). More hippocampal neurons in adult mice living in an enriched environment. Nature.

[35] Golmohammadi M.G., Blackmore D.G., Large B., Azari H., Esfandiary E., Paxinos G., Franklin K.B., Reynolds B.A., Rietze R.L. (2008). Comparative analysis of the frequency and distribution of stem and progenitor cells in the adult mouse brain. Stem Cells.

[36] Hermann A., Suess C., Fauser M., Kanzler S., Witt M., Fabel K., Schwarz J., Hoglinger G.U., Storch A. (2009). Rostro-caudal gradual loss of cellular diversity within the periventricular regions of the ventricular system. Stem Cells.

[37] Brazel C.Y., Limke T.L., Osborne J.K., Miura T., Cai J., Pevny L., Rao M.S. (2005). Sox2 expression defines a heterogeneous population of neurosphere-forming cells in the adult murine brain. Aging Cell.

[38] Doetsch F., Caille I., Lim D.A., Garcia-Verdugo J.M., Alvarez-Buylla A. (1999). Subventricular zone astrocytes are neural stem cells in the adult mammalian brain. Cell.

[39] Hermann A., Maisel M., Wegner F., Liebau S., Kim D.W., Gerlach M., Schwarz J., Kim K.S., Storch A. (2006). Multipotent neural stem cells from the adult tegmentum with dopaminergic potential develop essential properties of functional neurons. Stem Cells.

[40] Jaberi R., Mirsadeghi S., Kiani S. (2021). In vitro characterization of subventricular zone isolated neural stem cells, from adult monkey and rat brain. Mol. Biol. Rep..

[41] Wachs F.P., Couillard-Despres S., Engelhardt M., Wilhelm D., Ploetz S., Vroemen M., Kaesbauer J., Uyanik G., Klucken J., Karl C. (2003). High efficacy of clonal growth and expansion of adult neural stem cells. Lab. Investig..

[42] Frielingsdorf H., Schwarz K., Brundin P., Mohapel P. (2004). No evidence for new dopaminergic neurons in the adult mammalian substantia nigra. Proc. Natl. Acad. Sci. USA.

[43] Alonso G. (1999). Neuronal progenitor-like cells expressing polysialylated neural cell adhesion molecule are present on the ventricular surface of the adult rat brain and spinal cord. J. Comp. Neurol..

[44] Lois C., Alvarez-Buylla A. (1994). Long-distance neuronal migration in the adult mammalian brain. Science.

[45] Herrera D.G., Garcia-Verdugo J.M., Alvarez-Buylla A. (1999). Adult-derived neural precursors transplanted into multiple regions in the adult brain. Ann. Neurol..

[46] Seidenfaden R., Desoeuvre A., Bosio A., Virard I., Cremer H. (2006). Glial conversion of SVZ-derived committed neuronal precursors after ectopic grafting into the adult brain. Mol. Cell. Neurosci..

[47] Zigova T., Pencea V., Betarbet R., Wiegand S.J., Alexander C., Bakay R.A., Luskin M.B. (1998). Neuronal progenitor cells of the neonatal subventricular zone differentiate and disperse following transplantation into the adult rat striatum. Cell Transplant..

[48] Zhang R.L., Zhang L., Zhang Z.G., Morris D., Jiang Q., Wang L., Zhang L.J., Chopp M. (2003). Migration and differentiation of adult rat subventricular zone progenitor cells transplanted into the adult rat striatum. Neuroscience.

[49] Shetty A.K., Hattiangady B. (2016). Grafted Subventricular Zone Neural Stem Cells Display Robust Engraftment and Similar Differentiation Properties and Form New Neurogenic Niches in the Young and Aged Hippocampus. Stem Cells Transl. Med..

[50] Suhonen J.O., Peterson D.A., Ray J., Gage F.H. (1996). Differentiation of adult hippocampus-derived progenitors into olfactory neurons in vivo. Nature.

[51] Jamal A.L., Walker T.L., Waber Nguyen A.J., Berman R.F., Kempermann G., Waldau B. (2015). Transplanted Dentate Progenitor Cells Show Increased Survival in an Enriched Environment But Do Not Exert a Neurotrophic Effect on Spatial Memory Within 2 Weeks of Engraftment. Cell Transplant..

[52] Takahashi M., Palmer T.D., Takahashi J., Gage F.H. (1998). Widespread integration and survival of adult-derived neural progenitor cells in the developing optic retina. Mol. Cell. Neurosci..

[53] Hattiangady B., Shuai B., Cai J., Coksaygan T., Rao M.S., Shetty A.K. (2007). Increased dentate neurogenesis after grafting of glial restricted progenitors or neural stem cells in the aging hippocampus. Stem Cells.

[54] Brilli E., Reitano E., Conti L., Conforti P., Gulino R., Consalez G.G., Cesana E., Smith A., Rossi F., Cattaneo E. (2013). Neural stem cells engrafted in the adult brain fuse with endogenous neurons. Stem Cells Dev..

[55] Lie D.C., Dziewczapolski G., Willhoite A.R., Kaspar B.K., Shults C.W., Gage F.H. (2002). The adult substantia nigra contains progenitor cells with neurogenic potential. J. Neurosci..

[56] Shihabuddin L.S., Horner P.J., Ray J., Gage F.H. (2000). Adult spinal cord stem cells generate neurons after transplantation in the adult dentate gyrus. J. Neurosci..

[57] van Praag H., Christie B.R., Sejnowski T.J., Gage F.H. (1999). Running enhances neurogenesis, learning, and long-term potentiation in mice. Proc. Natl. Acad. Sci. USA.

[58] Franklin K.B., Paxinos G. (2012). The Mouse Brain in Stereotaxic Coordinates.

[59] Brandt M.D., Krüger-Gerlach D., Hermann A., Meyer A.K., Kim K.S., Storch A. (2017). Early Postnatal but Not Late Adult Neurogenesis Is Impaired in the Pitx3-Mutant Animal Model of Parkinson’s Disease. Front. Neurosci..

[60] Key G., Kubbutat M.H., Gerdes J. (1994). Assessment of cell proliferation by means of an enzyme-linked immunosorbent assay based on the detection of the Ki-67 protein. J. Immunol. Methods.

[61] Gerdes J., Lemke H., Baisch H., Wacker H.H., Schwab U., Stein H. (1984). Cell cycle analysis of a cell proliferation-associated human nuclear antigen defined by the monoclonal antibody Ki-67. J. Immunol..

[62] Scholzen T., Gerdes J. (2000). The Ki-67 protein: From the known and the unknown. J. Cell Physiol..

[63] Raedt R., Van Dycke A., Waeytens A., Wyckhuys T., Vonck K., Wadman W., Boon P. (2009). Unconditioned adult-derived neurosphere cells mainly differentiate towards astrocytes upon transplantation in sclerotic rat hippocampus. Epilepsy Res..

[64] Hermann A., Brandt M.D., Loewenbruck K.F., Storch A. (2010). “Silenced” polydendrocytes: A new cell type within the oligodendrocyte progenitor cell population?. Cell Tissue Res..

[65] Altman J. (1963). Autoradiographic investigation of cell proliferation in the brains of rats and cats. Anat. Rec..

[66] Reynolds B.A., Weiss S. (1992). Generation of neurons and astrocytes from isolated cells of the adult mammalian central nervous system. Science.

[67] Magavi S.S., Leavitt B.R., Macklis J.D. (2000). Induction of neurogenesis in the neocortex of adult mice. Nature.

[68] Bennett L., Yang M., Enikolopov G., Iacovitti L. (2009). Circumventricular organs: A novel site of neural stem cells in the adult brain. Mol. Cell. Neurosci..

[69] Lledo P.M., Merkle F.T., Alvarez-Buylla A. (2008). Origin and function of olfactory bulb interneuron diversity. Trends Neurosci..

[70] Chen X., Tolkovsky A.M., Herbert J. (2011). Cell origin and culture history determine successful integration of neural precursor transplants into the dentate gyrus of the adult rat. PLoS ONE.

[71] Gage F.H., Coates P.W., Palmer T.D., Kuhn H.G., Fisher L.J., Suhonen J.O., Peterson D.A., Suhr S.T., Ray J. (1995). Survival and differentiation of adult neuronal progenitor cells transplanted to the adult brain. Proc. Natl. Acad. Sci. USA.

[72] Kreisel T., Wolf B., Keshet E., Licht T. (2019). Unique role for dentate gyrus microglia in neuroblast survival and in VEGF-induced activation. Glia.

[73] Kreisel T., Frank M.G., Licht T., Reshef R., Ben-Menachem-Zidon O., Baratta M.V., Maier S.F., Yirmiya R. (2014). Dynamic microglial alterations underlie stress-induced depressive-like behavior and suppressed neurogenesis. Mol. Psychiatry.

[74] Gemma C., Bachstetter A.D. (2013). The role of microglia in adult hippocampal neurogenesis. Front. Cell. Neurosci..

[75] Alvarez-Dolado M., Pardal R., Garcia-Verdugo J.M., Fike J.R., Lee H.O., Pfeffer K., Lois C., Morrison S.J., Alvarez-Buylla A. (2003). Fusion of bone-marrow-derived cells with Purkinje neurons, cardiomyocytes and hepatocytes. Nature.

[76] Beyer F., Samper Agrelo I., Kury P. (2019). Do Neural Stem Cells Have a Choice? Heterogenic Outcome of Cell Fate Acquisition in Different Injury Models. Int. J. Mol. Sci..

