# Supplementary material for: Adult Neural Stem Cells from Midbrain Periventricular Regions Show Limited Neurogenic Potential after Transplantation into the Hippocampal Neurogenic Niche
Source: Cells. 2021 Nov 4;10(11):3021. doi: 10.3390/cells10113021 (PMC8616334; doi:10.3390/cells10113021)
Supplement: Supplementary file 1 [file cells-10-03021-s001.zip › cells-1437704-supplementary.pdf]

Supplementary Information

Adult neural stem cells from midbrain periventricular regions show limited neurogenic potential after transplantation into the hippocampal neurogenic niche

Mareike Fauser, Kai Loewenbrück, Johannes Rangnick, Moritz D. Brandt, Andreas Hermann, Alexander Storch

Supplementary Tables:

Supplementary Tables S1-S9. Statistical results of two-way mixed ANOVA.

Supplementary Figures:

Supplementary Figure S1: Subregional distribution of Sox2$^+$ neural stem cells and NeuroD1$^+$ immature neurons 7 days after transplantation.

Supplementary Figure S2: Subregional distribution of NG2$^+$ oligodendroglial progenitor cells 7 days after transplantation.

Supplementary Table S1: Statistics determined for the relative amounts of surviving GFP^+ cells after transplantation within the different hippocampal regions depending on graft origin (**Figure 2D**). Two-way mixed ANOVA showed significant main effects of hippocampal region on GFP^+ cell numbers (proportion of GFP^+ cells per region) overall ($F(1,35)=11.21$, $P=0.001$, with Greenhouse-Geisser correction). There was no significant interaction between hippocampal region and transplanted aNSC type ($PVR_{V\text{-}SVZ}$ vs. PVR_{MB}) in terms of GFP^+ cell survival ($F(1,35)=2.84$, $P=0.092$, with Greenhouse-Geisser correction). There was no significant main effect of transplanted aNSC type on GFP^+ cell survival ($F(1,29)=0.0$, $P=1.000$). Results of *post-hoc* t-tests with Bonferroni adjustment revealed the *P*-values as displayed in **(A)** for significances among the different hippocampal regions.

A

	**$PVR_{V\text{-}SVZ}$**	**PVR_{MB}**
**Subgranular Zone vs. Granular Zone**	**<0.001**	**<0.001**
**Subgranular Zone vs. Hilus**	0.082	1.000
**Granular Zone vs. Hilus**	1.000	**0.008**

Supplementary Table S2: Statistics determined for the relative amounts of proliferating GFP$^+$ cells (BrdU$^+$/GFP$^+$ cells) after transplantation within the different hippocampal regions depending on graft origin (**Figure 2F**). Two-way mixed ANOVA showed significant main effects of hippocampal region on BrdU$^+$/GFP$^+$ cells numbers (proportion of BrdU$^+$/GFP$^+$ cells per region) overall ($F(2,45)=5.30$, $P=0.012$, with Greenhouse-Geisser correction). There was no significant interaction between hippocampal region and transplanted aNSC type ($PVR_{V\text{-}SVZ}$ vs. PVR_{MB}) in terms of BrdU$^+$ cell amounts ($F(2,45)=0.79$, $P=0.441$, with Greenhouse-Geisser correction). There was no significant main effect of transplanted aNSC type on BrdU$^+$ cell amounts ($F(1.,27)=2.23$, $P=0.147$). Results of *post-hoc* t-tests with Bonferroni adjustment revealed the *P*-values as displayed in **(A)** for significances among the different hippocampal regions.

A

	$PVR_{V\text{-}SVZ}$	PVR_{MB}
**Subgranular Zone vs. Granular Zone**	1.000	1.000
**Subgranular Zone vs. Hilus**	**0.044**	0.151
**Granular Zone vs. Hilus**	0.186	0.409

Supplementary Table S3: Statistics determined for the total relative amounts of GFP$^+$/Sox2$^+$ cells after transplantation within the different hippocampal regions (SGZ, GZ) depending on graft origin (**Supplementary Figure S1A**). Two-way mixed ANOVA showed no significant main effect of hippocampal region on GFP$^+$/Sox2$^+$ cell numbers (proportion of GFP$^+$/Sox2$^+$ cells per region) overall ($F(1,19)=0.55$, $P=0.466$). There was no significant interaction between hippocampal region and transplanted aNSC type ($PVR_{V\text{-}SVZ}$ vs. PVR_{MB}) in terms of total Sox2$^+$ cell amounts ($F(1,19)=0.20$, $P=0.658$). There was no significant main effect of transplanted aNSC type ($PVR_{V\text{-}SVZ}$ vs. PVR_{MB}) on total GFP$^+$/Sox2$^+$ cell amounts ($F(1,19)=0.66$, $P=0.425$).

Supplementary Table S4: Statistics determined for BrdU labeling index of $GFP^+/Sox2^+$ cells after transplantation within the different hippocampal regions (SGZ, GZ) depending on graft origin (**Supplementary Figure S1B**). Two-way mixed ANOVA showed significant main effects of hippocampal region on $GFP^+/Sox2^+/BrdU^+$ cell numbers overall (F(1,16)=1.88, *P*<0.001). There was no significant interaction between hippocampal region and transplanted aNSC type ($PVR_{V\text{-}SVZ}$ vs. PVR_{MB}) in terms of $Sox2^+/BrdU^{+/-}$ cell amounts (F(1,16)=0.18, *P*=0.679). There was no significant main effect of transplanted aNSC type on $GFP^+/Sox2^+/BrdU^{+/-}$ cell amounts (F(1,16)=0.42, *P*=0.528).

Supplementary Table S5: Statistics determined for the relative amounts of surviving GFP^+ cells after transplantation within the different hippocampal regions (SGZ, GZ) depending on running of host animal. Two-way mixed ANOVA showed significant main effects of hippocampal region on GFP^+ cell numbers (proportion of GFP^+ cells per region) overall (F(2,13)=40.81, *P*<0.001, with Greenhouse-Geisser correction). There was no significant interaction between hippocampal region and running group (running vs. control) in terms of GFP^+ cell survival (F(2,13)=1.58, *P*=0.239, with Greenhouse-Geisser correction). There was no significant main effect of running group (running vs. control) on GFP^+ cell survival (F(1,9)=0.05, *P*=0.828). Results of *post-hoc* t-tests with Bonferroni adjustment revealed the *P*-values as displayed in **(A)** for significances among the different hippocampal regions.

A

	**Runners**	**Controls**
**Subgranular Zone vs. Granular Zone**	**0.001**	**0.002**
**Subgranular Zone vs. Hilus**	0.898	1.000
**Granular Zone vs. Hilus**	**0.004**	**0.008**

Supplementary Table S6: Statistics determined for the BrdU labeling index of GFP^+ cells after transplantation of $PVR_{V\text{-}SVZ}$ aNSC within the different hippocampal regions (SGZ, GZ) depending on running of host animal. Two-way mixed ANOVA showed no significant main effect of hippocampal region on $BrdU^+/GFP^+$ cells numbers (proportion of $BrdU^+/GFP^+$ cells per region) overall ($F(2,12)=3.38$, $P=0.076$). There was no significant interaction between hippocampal region and running group (running vs. control) in terms of $BrdU^+$ cell amounts ($F(2,12)=0.1.47$, $P=0.266$). There was no significant main effect of running group type on $BrdU^+$ cell amounts ($F(1,7)=0.05$, $P=0.828$).

Supplementary Table S7: Statistics determined for the relative amounts of $GFP^+/Sox2^+$ cells after transplantation within the different hippocampal regions (SGZ, GZ) depending on running of host animals. Two-way mixed ANOVA showed no significant main effect of hippocampal region on $GFP^+/Sox2^+$ cell numbers (proportion of $GFP^+/Sox2^+$ cells per region) overall ($F(1,6)=0.87$, $P=0.387$). There was no significant interaction between hippocampal region and running groups (runners vs. controls) in terms of total $Sox2^+$ cell amounts ($F(1,6)=0.1.09$, $P=0.337$). There was no significant main effect of running group (runners vs. controls) on total $GFP^+/Sox2^+$ cell amounts ($F(1,6)=0.03$, $P=0.877$).

Supplementary Table S8: Statistics determined for the relative total amounts of GFP$^+$/NeuroD1$^+$ cells after transplantation within the different hippocampal regions depending on graft origin (**Supplementary Figure S1C**). Two-way mixed ANOVA showed significant main effects of hippocampal region on GFP$^+$/NeuroD1$^+$ cell numbers (proportion of GFP$^+$/NeuroD1$^+$ cells per region) overall ($F(1,11)=16.49$, $P=0.002$). There was no significant interaction between hippocampal region and transplanted aNSC type ($PVR_{V\text{-}SVZ}$ vs. PVR_{MB}) in terms of total NeuroD1$^+$ cell amounts ($F(1,11)=1.37$, $P=0.266$). There was no significant main effect of transplanted aNSC type ($PVR_{V\text{-}SVZ}$ vs. PVR_{MB}) on total GFP$^+$/NeuroD1$^+$ cell amounts ($F(1,19)=0.31$, $P=0.588$). Results of *post-hoc* t-tests the *P*-values as displayed in **(A)** for significances among the different hippocampal regions. Bold values indicate significant differences.

A

	**$PVR_{V\text{-}SVZ}$**	**PVR_{MB}**
**GFP$^+$/NeuroD1$^+$ in SGZ vs. GFP$^+$/NeuroD1^{+-} in GZ**	**0.040**	**0.007**

Supplementary Table S9: Statistics determined for the relative total amounts of GFP$^+$/NG2$^+$ cells after transplantation within the different hippocampal regions depending on graft origin (**Supplementary Figure S2B**). Two-way mixed ANOVA showed significant main effects of hippocampal region on GFP$^+$/NG2$^+$ cell numbers (proportion of GFP$^+$/NG2$^+$ cells per region) overall ($F(1,7)=0.06$, $P=0.817$). There was no significant interaction between hippocampal region and transplanted aNSC type ($PVR_{V\text{-}SVZ}$ vs. PVR_{MB}) in terms of total NG2$^+$ cell amounts ($F(1,7)=2.74$, $P=0.142$). There was no significant main effect of transplanted aNSC type ($PVR_{V\text{-}SVZ}$ vs. PVR_{MB}) on total GFP$^+$/NG2$^+$ cell amounts ($F(1,7)=1.51$, $P=0.259$).

Supplementary Figures & Legends to Supplementary Figures

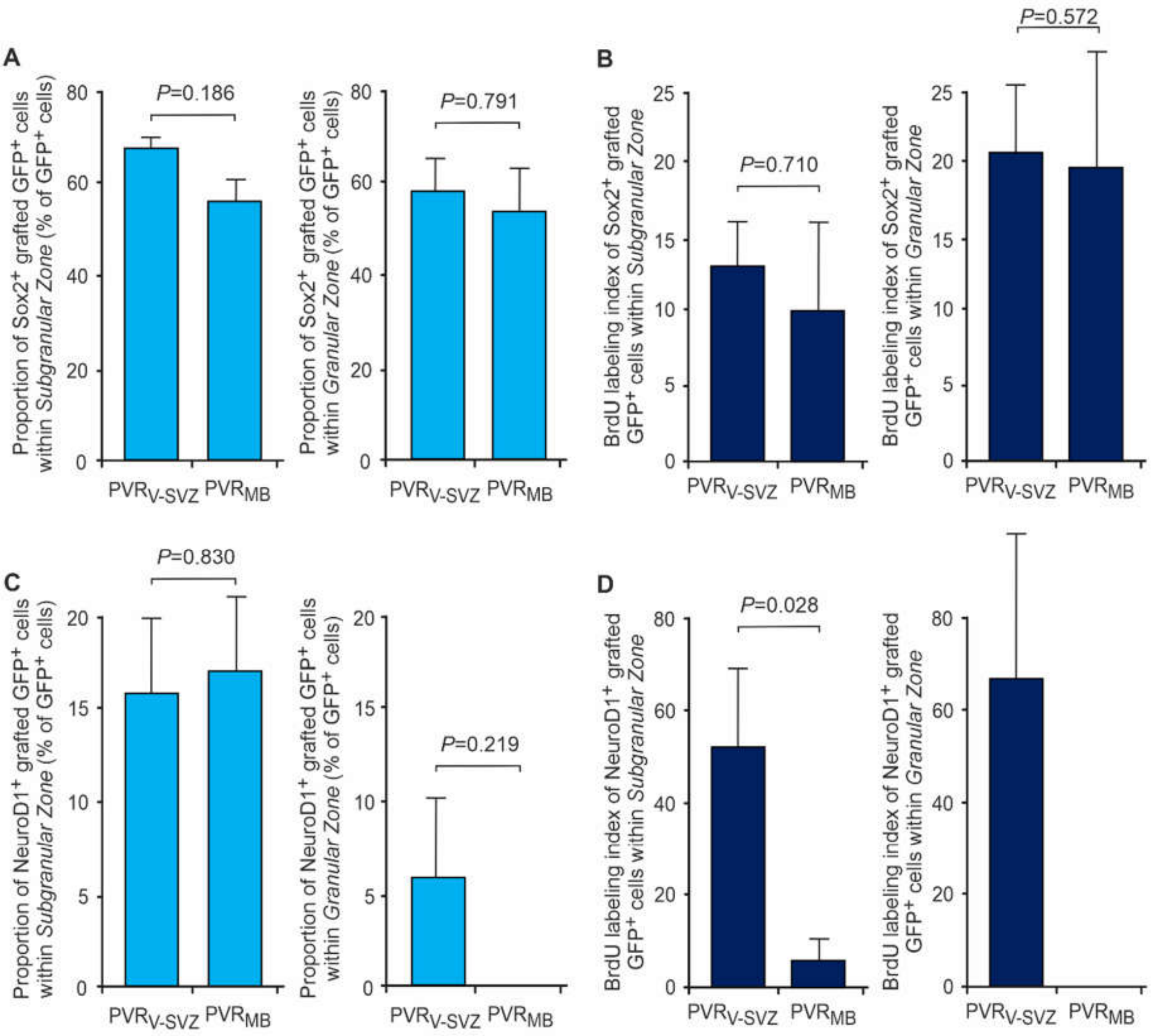

Supplementary Figure S1: Subregional distribution of Sox2^{+} neural stem cells and NeuroD1^{+} immature neurons 7 days after transplantation.

(A,B) Relative Sox2^{+} cell counts normalized to total GFP^{+} grafted cell counts within the two main subregions of the DG, namely the SGZ and the GZ. Sox2^{+} cell counts as well as BrdU labeling index of Sox2^{+} cells counts did not differ between the two grafts (PVR$_{V\text{-}SVZ}$ vs. PVR$_{MB}$)

in both DG subregions ($P \geq 0.05$, two-way mixed ANOVA with hippocampal region [SGZ, GS] and transplanted aNSC type [$PVR_{V\text{-}SVZ}$ vs. PVR_{MB}] as independent variables; see **Supplementary Table S3,S4** for statistical results). *P*-values are from *post-hoc* t-tests with Bonferroni adjustment for multiple comparisons ($PVR_{V\text{-}SVZ}$ n=7; PVR_{MB} n=21). **(C,D)** Relative $NeuroD1^{+}$ cell counts normalized to total GFP^{+} grafted cell counts within SGZ and GZ. $NeuroD1^{+}$ cells counts varied significantly between the subregions, but did not differ between the two grafts in both DG subregions (see **Supplementary Table S8** for statistical results). BrdU labeling index of $NeuroD1^{+}$ cells differed between the grafts in the SGZ. *P*-values are from *post-hoc* t-tests with Bonferroni adjustment for multiple comparisons for $NeuroD1^{+}$ cell counts and unpaired t-test für BrdU labeling index in SGZ ($PVR_{V\text{-}SVZ}$ n=8; PVR_{MB} n=7).

Abbreviations: $PVR_{V\text{-}SVZ}$ - periventricular region of the ventricular-subventricular zone of the lateral wall of the lateral ventricles; PVR_{MB} – periventricular region of the midbrain; aNSC – adult neural stem cell; SGZ - subgranular zone; GZ - granular zone; GFP - green fluorescent protein; BrdU - 5’-bromo-2’-desoxyuridine; DG - dentate gyrus.

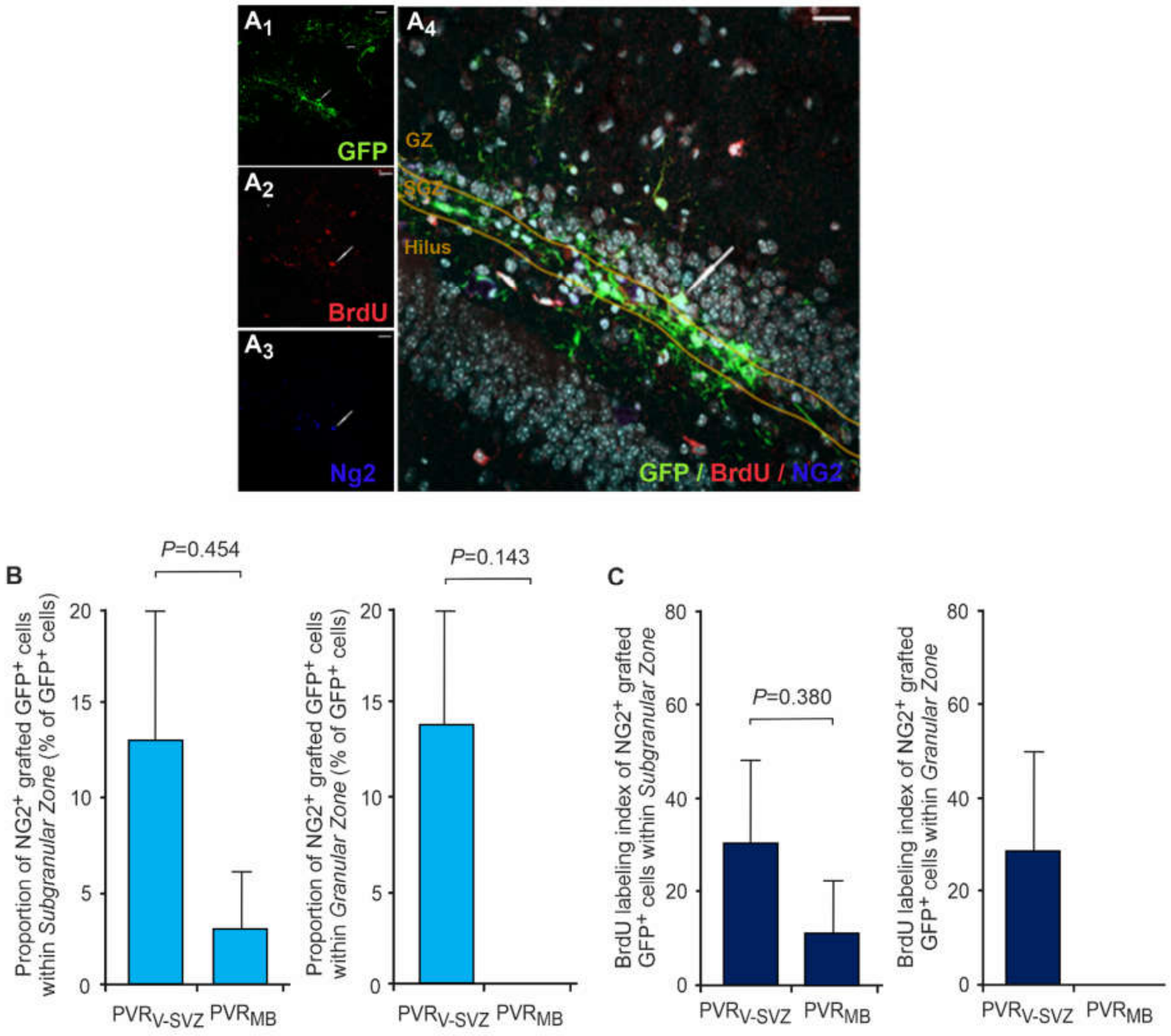

Supplementary Figure S2: Subregional distribution of NG2$^+$ oligodendroglial progenitor cells 7 days after transplantation.

(A) Representative triple fluorescence immunostaining of a $PVR_{V\text{-}SVZ}$ graft within the DG (the arrow illustrates a $GFP^+/BrdU^+/NG2^+$ grafted polydendrocyte; **A**$_4$). GFP identifies transplanted cells, (green, **A**$_1$), BrdU (red, **A**$_2$) indicates cells which proliferated after transplantation and NG2 serves as a marker for polydendrocytes (blue, **A**$_3$). Scale bars, 10 µm. **(B)** Relative NG2$^+$ cell counts normalized to total GFP$^+$ grafted cell counts within SGZ and GZ. NG2$^+$ and BrdU

labeling index of $NG2^+$ polydendrocyte counts did not differ between the two grafts in both DG subregions (see **Supplementary Table S9** for statistical results). *P*-values are from *post-hoc* t-tests with Bonferroni adjustment for multiple comparisons for $NG2^+$ cell counts and unpaired t-test für BrdU labeling index in SGZ ($PVR_{V\text{-}SVZ}$ n=7; PVR_{MB} n=8).

Abbreviations: $PVR_{V\text{-}SVZ}$ - periventricular region of the ventricular-subventricular zone of the lateral ventricles; PVR_{MB} – periventricular region of the midbrain; aNSC – adult neural stem cell; SGZ - subgranular zone; GZ - granular zone; GFP - green fluorescent protein; BrdU - 5'-bromo-2'-desoxyuridine.
